# AI Tool Use Among Osteopathic Medical Students: Pilot Digital Diary Study

**DOI:** 10.2196/96895

**Published:** 2026-09-01

**Authors:** Carinne Brody, Seth Schwindt, Achint Thakur, Pieter von Steinbergs

**Affiliations:** 1 Public Health Program and Office of Research, Touro University California, 1310 Club Drive, Vallejo, CA, 94592, United States, 1 7076388533

**Keywords:** artificial intelligence, medical education, diary study, ChatGPT, osteopathy, pilot study

## Abstract

**Background:**

AI is increasingly integrated into medical education, offering new ways for students to acquire knowledge and support clinical reasoning. However, the extent, patterns, and implications of AI use among medical students remain incompletely understood. Prior studies have relied on retrospective surveys that are susceptible to recall bias and have not quantified AI use as a proportion of total study time.

**Objective:**

This pilot study aimed to quantify real-time AI use among medical students, including the proportion of study time devoted to AI, and how use varies by training stage and engagement style (active vs passive). Active use was defined as iterative, bidirectional engagement; passive use was defined as unidirectional consultation with limited interrogation.

**Methods:**

This longitudinal observational cohort study recruited medical students from 2 osteopathic medical schools (April-May 2025) to complete a baseline survey and 7 digital diary entries over a 21-day period, delivered via automated SMS every 3 days. Students reported total study time, AI use time, tools used, and purposes of use. The data were analyzed using Stata 19. Multiple linear regression models examined associations between AI use (total minutes and percentage of study time) and key variables, and a mixed-effects model using diary-level data with a random intercept per student addressed within-person variability across entries.

**Results:**

A total of 71 of 1332 (response rate: 5.3%) eligible students completed the baseline survey (mean age 26.6, SD 2.8 y; n=39, 55% identified as men; n=32, 45% identified as women; n=55, 77% preclinical). On average, students reported using AI tools during 19% of their total study time (mean 35.8 of 185.6 min per diary, SD 35.8 min). The most used tool was ChatGPT (n=63, 89%), followed by Google Gemini (n=22, 31%). Clinical-phase students (MS3-MS4) used AI significantly more than preclinical students (MS1-MS2), with an adjusted increase of 19% (*P*=.003). Students classified as active users spent significantly more total time using AI than passive users (*P*=.002). Across groups, AI use was primarily passive, including simplifying complex concepts, answering practice questions, and generating summaries. In the multilevel models, preclinical students reported significantly lower AI use than clinical-phase students (*P*=.02). The intraclass correlation coefficient was 0.47 (95% CI 0.34‐0.60).

**Conclusions:**

Although preliminary, these findings suggest that medical students are incorporating AI into a substantial proportion of their study time, with greater use among clinical trainees and active users. Despite this, most use remains passive. Given mixed evidence on AI’s impact on deep learning, further research on learning outcomes is needed. Institutions may consider providing guidance on responsible AI use, including critical evaluation and verification of outputs. The digital diary methodology offers a practical approach for capturing real-time AI use and may inform future educational research and intervention design.

## Introduction

The integration of AI into medical education is rapidly transforming the way students acquire knowledge, develop clinical reasoning skills, and engage in lifelong learning [1]. In higher education, AI-powered tools, such as machine learning algorithms, large language models, and automated tutoring programs, are increasingly being used to enhance traditional learning methods [2].

Within medical education specifically, AI tools are being used to support self-directed learning, concept review, and examination preparation. ChatGPT has been reported to perform at or above the median performance among 276,779 student test takers on the Medical College Admission Test (MCAT) and has performed at or near the passing threshold for all 3 steps of the United States Medical Licensing Examination (USMLE) [3,4]. These capabilities have driven rapid student adoption, with surveys suggesting between 52% and 90% of medical students in various settings have used AI tools for studying [5-8]. AI is also influencing professional development, with 43% of residency applicants planning to use AI for personal statements [9].

The ethical discussions surrounding AI use by medical students are still developing, emphasizing the importance of understanding its limitations, verifying sources, ensuring Health Insurance Portability and Accountability Act (HIPAA) compliance, and maintaining academic integrity [10]. Critically, there is limited guidance specific to student use of AI during training, as distinct from clinical or research contexts [11,12].

Existing evidence suggests that medical students have a range of knowledge of AI tools. A 2023 systematic review found that students had generally positive attitudes toward AI in medicine, but most had low knowledge and limited skills [13]. By 2024, high knowledge and high usage (70%) were reported among 102 US medical students, with AI exposure associated with greater trust in clinical AI applications [14].

However, how students engage with large language models during studying remains incompletely characterized. A survey of 415 students from 28 US medical schools found 52% used AI tools for schoolwork, primarily for concept explanation during preclerkship and diagnostic assistance during clerkship [15]. While informative, these studies relied on retrospective recall, did not quantify the proportion of study time devoted to AI, and did not capture real-time patterns of use. To our knowledge, no prior study has used a prospective diary methodology to measure AI use as a percentage of total study time.

Digital diaries, also called ecological momentary assessment or experience sampling, are a validated method for prospective data collection that reduce recall bias by capturing experiences in or near real time [16,17]. Unlike learning portfolios, which are reflective and often summative, digital diaries capture ongoing behaviors at regular intervals with minimal retrospective reconstruction.

This study assessed the adoption and use of AI tools by medical students during studying and schoolwork through a longitudinal digital diary to capture accurate accounts of the nature and frequency of AI use. These findings may inform institutional policies, curriculum design, and preparation of future health care professionals to navigate AI in clinical practice.

## Methods

### Study Design and Setting

**Figure 1.**
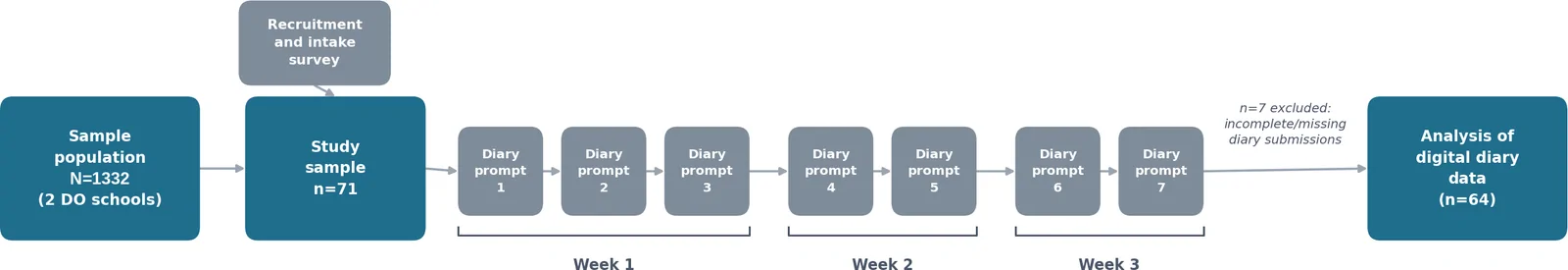
Pilot digital diary study design. DO: doctor of osteopathy.

This was a longitudinal observational cohort study using a digital diary methodology. The study was conducted at Touro University California (TUC) and Touro University Nevada (TUN), both accredited colleges of osteopathic medicine in the United States. This study is reported in accordance with the STROBE (Strengthening the Reporting of Observational Studies in Epidemiology) statement for cohort studies; the completed checklist is provided in Checklist 1. Recruitment spanned from April 10 to 28, 2025. Each participant began a 21-day digital diary upon completing the baseline survey; the final diary entry was collected on May 19, 2025. The study consisted of a one-time intake survey followed by a 21-day digital diary phase (Figure 1). Digital diaries have been shown to be a valid method of collecting responses while minimizing recall bias [16]. Participants received survey prompts 3 times per week for 3 weeks—an approach that balanced reducing recall bias and maximizing participation [17].

No formal power calculation was conducted; the sample represents a convenience sample of all willing enrollees during the recruitment period, consistent with the pilot or exploratory aims of the study.

### Participants and Recruitment

All currently enrolled doctor of osteopathy (DO) students from both campuses were eligible to participate. Recruitment was conducted through email announcements distributed via institutional listservs. A total of 1332 students were invited to participate: 601 via the TUC listserv and 731 via the TUN listserv. Interested students received an electronic informed consent form and were enrolled in the study upon providing consent. Of those invited, 71 (5.3%) students expressed interest, consented, and were enrolled; there were no withdrawals and 7 losses to follow-up during the 21-day diary period. The study recruitment period spanned from April 10 to 28, 2025. Each participant was on their own 21-day digital diary schedule beginning upon the completion of their intake survey. The final digital diary entry was collected on May 19, 2025.

To assess representativeness, we compared our sample’s gender composition with DO program-specific institutional enrollment data. Women comprise approximately 54% of DO students at TUC and 47% at TUN. Weighting these benchmarks by our sample’s campus composition (76% TUC, 24% TUN) yields an expected population value of 52.3% women, compared with 45.1% (32/71) among enrolled participants; this difference was not statistically significant (*z*=−1.22; *P*=.22)

### Data Collection

Following informed consent, participants completed the baseline intake survey via Google Forms. The survey collected demographic information (age, self-reported gender [options: man, woman, nonbinary, other, and prefer not to say], year in training, campus), prior exposure to AI tools, perceived impact of AI on learning and clinical reasoning, and self-reported frequency and use cases of AI tools. The survey is available as a supplementary file (Multimedia Appendix 1).

After completing the intake survey, participants received a brief digital survey prompt every 3 days over a 21-day period (7 total entries). Surveys were delivered via automated SMS using SimpleTexting [18]. Each diary asked students to report (1) total minutes spent studying in the past 72 hours, (2) minutes using AI tools, (3) which tools were used, and (4) purposes of use (eg, concept explanation, summarization, question generation). “Total study time” was defined as self-reported minutes spent on academic activities (reading, reviewing notes, practice questions, school-related tasks) during the prior 72 hours. All responses were stored in a secure, password-protected cloud folder.

### Data Analysis

The data were analyzed using Stata 19 [19]. Descriptive statistics summarized all baseline variables. Digital diary data were aggregated to generate individual-level scores representing frequency and type of AI use. Subgroup analyses examined differences by year in training (preclinical vs clinical), gender, age, campus, and active versus passive use. Seven students who completed the intake survey did not submit any diary entries and were excluded from diary-based analyses (final analytic sample n=64). Completers and noncompleters did not differ significantly on measured demographic characteristics, suggesting exclusion is unlikely to have introduced systematic bias.

Active and passive use were defined a priori based on the cognitive engagement literature, drawing on emerging conceptual work on active versus passive AI engagement [20,21]. Active use was defined as iterative, bidirectional engagement with AI tools requiring critical appraisal and integration of outputs (eg, iterative prompting for clinical reasoning, evaluating competing diagnoses). Passive use was defined as unidirectional consultation with limited interrogation of outputs (eg, requesting a summary or explanation without follow-up).

Bivariate analyses (2-tailed *t* tests, chi-square tests) assessed group differences. Multiple linear regression examined the relationship between AI use (percentage of study time; total minutes) and key predictors, adjusting for age, gender, year in training, and active or passive use. Effect sizes for bivariate comparisons were calculated as Cohen *d* (small: *d*≥0.20, medium: *d*≥0.50, large: *d*≥0.80).

Regression analyses were conducted using listwise deletion. Effect sizes for regression predictors were calculated as Cohen *f*², computed as the difference in *R*² between the full model and a reduced model excluding each predictor, divided by 1 minus the full model *R*² (small: *f*²≥0.02, medium: *f*²≥0.15, large: *f*²≥0.35).

To directly address the repeated-measures structure of the diary data, we conducted mixed-effects (multilevel) regression models using diary-level rather than person-aggregated data, with a random intercept for each student to account for the nonindependence of repeated diary entries.

### Ethical Considerations

This study was approved by the TUC Institutional Review Board (IRB; TUC IRB application M-1425). All participants provided written informed consent. The study adhered to the Declaration of Helsinki. All research team members completed Collaborative Institutional Training Initiative (CITI) training. No identifying information was collected, and data were stored securely in compliance with federal data protection standards. As an incentive, students who completed the study were entered in a raffle for twenty-eight US $25 Visa gift cards and four US $50 Target gift cards.

## Results

### Participant Characteristics

**Figure 2.**
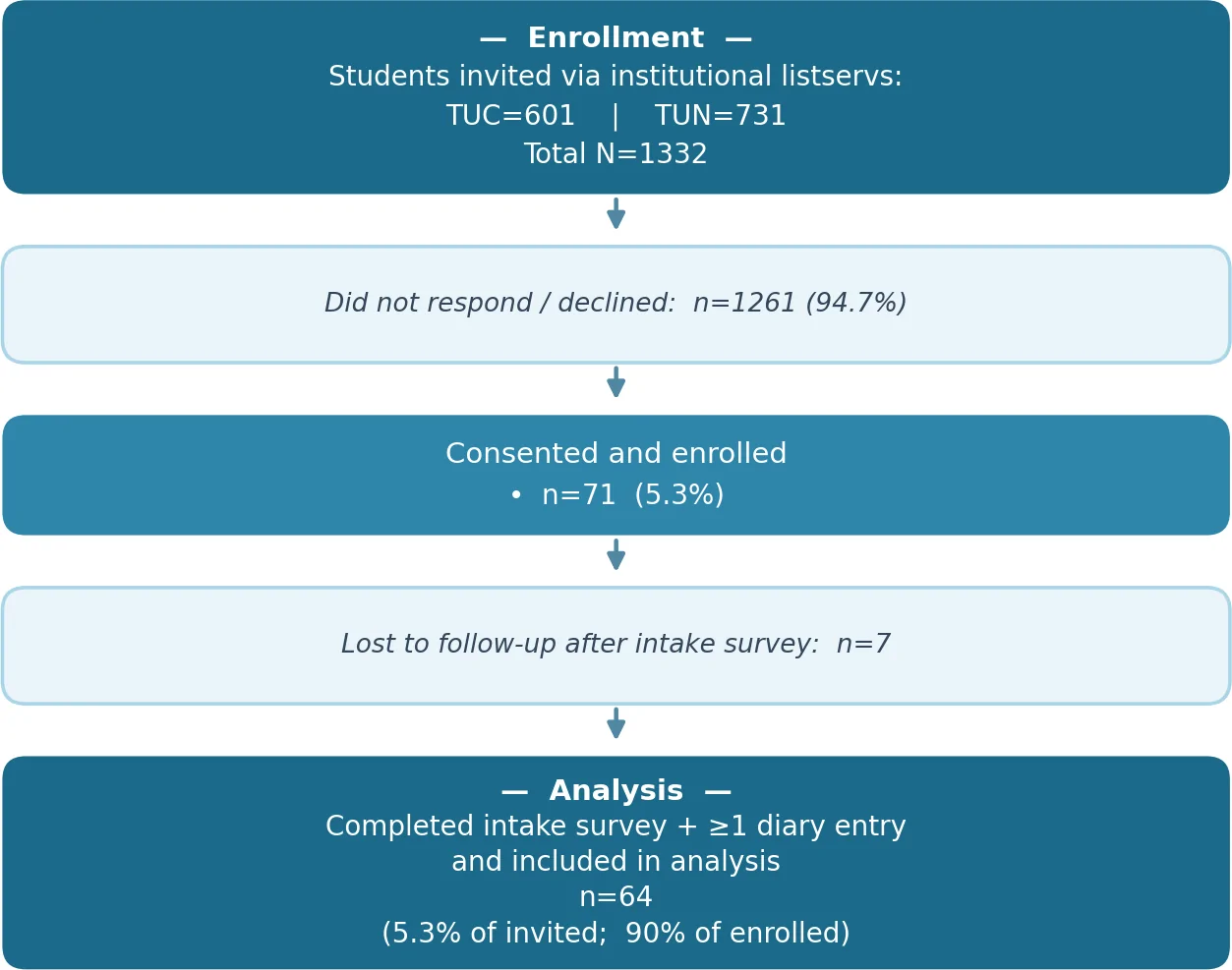
CONSORT (Consolidated Standards of Reporting Trials)-style participant flow. TUC: Touro University California; TUN: Touro University Nevada.

Of 1332 students invited via institutional listservs (601 at TUC; 731 at TUN), 71 (5.3%) consented and enrolled. No participants withdrew, while 7 did not complete any diaries. A total of 71 medical students completed the intake survey (Figure 2). The average age was 26.6 (SD 2.8) years. Among respondents, 39 (55%) identified as men and 32 (45%) identified as women. Students from all class years participated: 55 (77%) were in preclinical years MS1 or MS2, and 16 (23%) were in clinical years MS3, MS4, or research year. A total of 54 students (76%) were from TUC and 17 (24%) were from TUN. Of these, 32 completed all 7 diary entries, 26 completed 4 to 6 entries, and 7 completed 1 to 3 entries, and 7 completed 0 entries. Total diary entries received were as follows: 381 of 497 possible (71×7), representing 76.7% completion.

Regarding prior AI exposure, 31 (44%) students reported moderate prior exposure, 29 (41%) minimal exposure, 7 (10%) extensive exposure, and 4 (6%) no prior exposure. When asked whether AI tools should be formally incorporated into the curriculum, 43 (61%) students somewhat or strongly agreed, and 28 (39%) somewhat or strongly disagreed.

At baseline, 64 (90%) students reported having used AI tools for studying or schoolwork. Of those, 63 (98%) somewhat or strongly agreed that AI was positively impacting their learning, and 1 (2%) somewhat disagreed. Regarding clinical reasoning, 53 students (83%) somewhat or strongly agreed that AI was positively impacting their clinical reasoning, and 11 (17%) somewhat disagreed (Table 1).

**Table 1.** Demographic and baseline characteristics of medical students enrolled in a 21-day longitudinal digital diary study of AI tool use, Touro University California and Touro University Nevada, April-May 2025 (n=71).

Characteristic	Values
Age (y), mean (SD)	26.6 (2.8)
Gender, n/N (%)	
Man	39/71 (55)
Woman	32/71 (45)
Year in training, n/N (%)	
Preclinical (MS1-MS2)	55/71 (77)
Clinical (MS3-MS4)	16/71 (23)
Prior AI exposure, n/N (%)	
Extensive	7/71 (10)
Moderate	31/71 (44)
Minimal	29/71 (41)
None	4/71 (6)
AI should be in curriculum, n/N (%)	
Somewhat or strongly agree	43/71 (61)
Somewhat or strongly disagree	28/71 (39)
Ever used AI for studying, n/N (%)	
Yes	64/71 (90)
AI positively impacts learning, n/N (%)	
Agree	63/64 (98)
Disagree	1/64 (2)
AI positively impacts clinical reasoning, n/N (%)	
Agree	53/64 (83)
Disagree	11/64 (17)

### AI Use During Studying

The average time spent studying was 185.6 (SD 89.7 minutes per diary over the 3-week period. The average time using AI tools was 35.8 (SD 35.8 minutes, representing 19% of overall study time.

Specific AI tools used over the 3-week period are reported in Table 2. The most commonly used tool was ChatGPT (63/71, 89%), followed by Google Gemini (22/71, 31%), AMBOSS AI (11/71, 16%), Notebook LM (6/71, 9%), Open Evidence (5/71, 7%), UpToDate AI (5/71, 7%), and DeepSeek (3/71, 4%).

Students reported using AI for a variety of purposes (Table 2). Passive uses were most common: simplified explanations of difficult concepts (53/71, 75%), learning about complex medical topics (51/71, 72%), answering practice questions (37/71, 52%), creating disease or treatment summaries (29/71, 41%), and summarizing lecture notes (26/71, 37%). Active uses included generating mnemonics (25/71, 35%), interpreting lab results or imaging findings (22/71, 31%), simulating patient interactions (19/71, 27%), creating study schedules (17/71, 24%), generating flowcharts or algorithms (9/71, 13%), and using spaced repetition techniques (7/71, 10%).

**Table 2.** AI tools used and purposes of use reported by medical students during a 21-day digital diary study of AI use during studying, Touro University California and Touro University Nevada, April-May 2025 (n=65).

AI tools used and purposes of use	Values
AI tools and types of use	
Average study time per diary (min), mean (SD)	185.6 (89.7)
Average AI use time per diary (min), mean (SD)	35.8 (35.8)
Percentage of study time using AI	19
AI tools used, n/N (%)	
ChatGPT	63/71 (89)
Google Gemini	22/71 (31)
AMBOSS AI	11/71 (16)
Notebook LM	6/71 (9)
Open Evidence	5/71 (7)
UpToDate AI	5/71 (7)
DeepSeek	3/71 (4)
Passive uses, n/N (%)	
Simplifying explanations of difficult concepts	53/71 (75)
Learning about complex medical topics	51/71 (72)
Answering practice questions	37/71 (52)
Creating summaries of diseases or treatments	29/71 (41)
Summarizing lecture notes or textbooks	26/71 (37)
Active uses, n/N (%)	
Generating mnemonics or memory aids	25/71 (35)
Interpreting lab results or imaging findings	22/71 (31)
Simulating patient interactions or clinical scenarios	19/71 (27)
Creating study schedules or plans	17/71 (24)
Generating flowcharts or algorithms	9/71 (13)
Spaced repetition or active recall techniques	7/71 (10)

### Diary Trends

**Figure 3.**
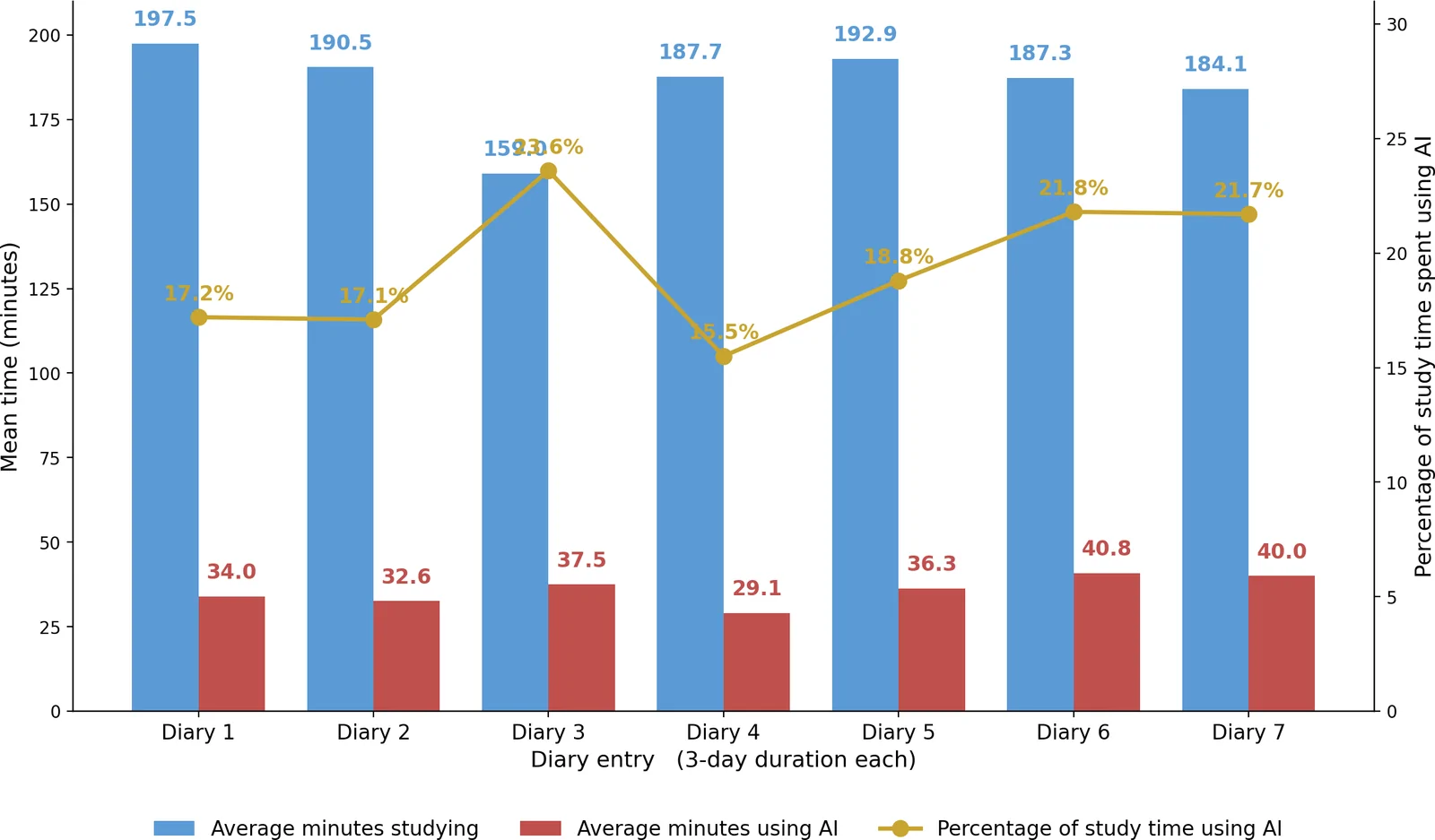
Average minutes spent studying and using AI tools across 7 diary entries over a 21-day digital diary study of medical students at Touro University California and Touro University Nevada, April-May 2025 (n=71).

Participants’ average study time and AI use were tracked over 7 diary days (Figure 3). Study time ranged from 159.0 minutes (diary 3) to 197.5 minutes (diary 1). AI use ranged from 29.1 minutes (diary 4) to 40.8 minutes (diary 6). The proportion of study time spent using AI ranged from 16% to 24% across diary days.

**Figure 4.**
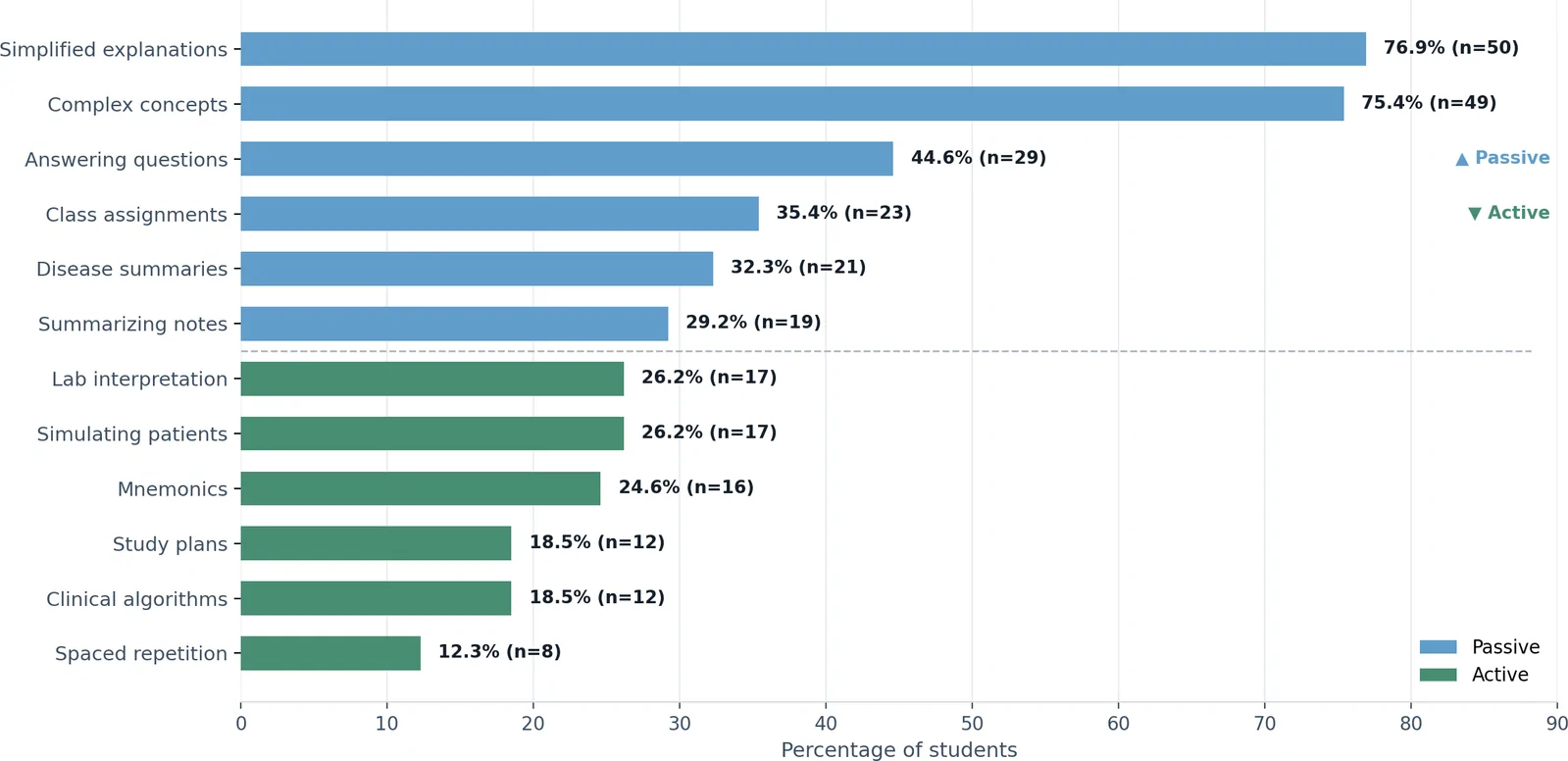
AI tool use by type and engagement category among medical students (n=65).

Among the 65 students who completed at least 1 diary entry, the most commonly reported passive uses of AI were simplified explanations of difficult concepts (n=50, 77%) and learning about complex concepts (n=49, 75%), followed by answering questions (n=29, 45%), class assignments (n=23, 35%), disease summaries (n=21, 32%), and summarizing notes (n=19, 29%). Active use was less common across all categories, with lab interpretation and simulating patients being the most frequently reported active uses (n=17 each, 26%), followed by mnemonics (n=16, 25%), study plans and clinical algorithms (n=12 each, 19%), and spaced repetition (n=8, 12%). The distribution of active and passive use types is shown in Figure 4.

### Subgroup Analyses

There were no significant differences in AI use percentage by gender (*P*=.14), age (*P*=.16), and campus (*P*=.41). A significant difference was observed by year in training: third- and fourth-year students reported using AI during 31% (127.5 min) of their study time, compared to 16% (145.2 min) among first- and second-year students (*P*=.003). There were no significant differences in AI total minutes by gender (*P*=.39), age (*P*=.93), and campus (*P*=.17). A significant difference was observed by year in training: third- and fourth-year students reported using AI during 31% (132 min) of their study time, compared to 16% (155 mins) among first- and second-year students (*P*=.003). The Cohen *d* for year in school on percent AI study (*d*=0.97) indicates a large effect (Table 3).

**Table 3.** Bivariate analysis of AI use (percentage of study time and total minutes) by demographic and engagement subgroups among medical students at Touro University California (TUC) and Touro University Nevada (TUN), April-May 2025 (n=65).

Variable	Percentage of study time using AI			Total minutes using AI		
	Mean (SD)	*P* value	Cohen *d*^a^ (95% CI)	Mean (SD)	*P* value	Cohen *d*^a^ (95% CI)
Gender		.14	−0.39 (−0.90 to 0.13)		.39	−0.22 (−0.72 to 0.28)
Woman	16.3 (11.4)			125.7 (156.4)		
Man	22.5 (20.7)			162.8 (186.6)		
Year in school		.003^b^	0.97 (0.33 to 1.61)		.73	−0.10 (−0.70 to 0.49)
Preclinical (MS1 or MS2)	15.8 (11.2)			145.2 (167.3)		
Clinical (MS3 or MS4)	30.5 (25.1)			127.5 (182.2)		
Age group (y)		.16	−0.37 (−0.88 to 0.15)		.93	−0.02 (−0.52 to 0.47)
≤26	16.3 (12.3)			139.6 (176.6)		
>26	22.2 (19.8)			143.6 (162.8)		
Campus		.41	0.25 (−0.35 to 0.85)		.17	0.39 (−0.17 to 0.95)
TUC	19.9 (16.5)			158.9 (156.9)		
TUN	15.8 (15.5)			92.6 (196.7)		
Active use		.48	0.10 (−0.41 to 0.60)		.007^b^	−0.64 (−1.14 to −0.14)
No	21.5 (25.4)			58.5 (60.2)		
Yes	18.1 (11.5)			178.9 (189.2)		

a Cohen *d* benchmarks: small, ≥0.20; medium, ≥0.50; large, ≥0.80. Negative values reflect group coding direction and should be interpreted as absolute magnitudes.

b *P*<.05.

### Regression Analyses

Two multivariable linear regression models were conducted to examine predictors of AI use (Tables 4 and 5). In the first model predicting the percentage of study time spent using AI (n=60), clinical-year students reported a significantly higher proportion of study time using AI compared with preclinical students (adjusted β=−16.98, 95% CI −27.90 to −6.07; *P*=.003), representing a medium effect size (Cohen *f*²=0.18). Age, active use classification, and campus were not significant predictors (all *P*>.10), with negligible to small effect sizes. The full model explained 18% of the variance in AI study time proportion (*R*²=0.18, Cohen *f*²=0.23).

**Table 4.** Multiple linear regression predicting percentage of study time using AI tools among medical students at Touro University California (TUC) and Touro University Nevada (TUN), April-May 2025 (n=60)^a^.

Predictor	β (SE; 95% CI)	*P* value	Cohen *f*²	Effect size
Year of training (preclinical ref.)	−16.98 (5.45; −27.90 to −6.07)	.003^b^	0.18	Medium
Age group (y; >27 vs <27)	0.54 (4.34; −8.17 to 9.25)	.90	0	Negligible
Active use (vs passive)	2.99 (4.13; −5.29 to 11.27)	.47	0.01	Negligible
Campus (TUN vs TUC)	−7.37 (4.93; −17.26 to 2.51)	.14	0.04	Small
Full model	—^c^	.02	0.23	Medium

a The analysis included 60 participants due to listwise deletion of cases with missing values on percent of AI use (n=5) or predictor variables (n=1).

b *P*<.05 indicates statistical significance.

c Not available.

**Table 5.** Multiple linear regression predicting total minutes of AI use during studying among medical students at Touro University California (TUC) and Touro University Nevada (TUN), April-May 2025 (n=64)^a^.

Predictor	β (SE; 95% CI)	*P* value	Cohen *f*²	Effect size
Year of training (preclinical ref.)	−34.48 (57.28; −149.10 to 80.13)	.61	0.01	Negligible
Age group (y; >27 vs <27)	−3.44 (46.12; −95.73 to 88.86)	.93	0	Negligible
Active use (vs passive)	108.42 (42.63; 23.11 to 193.74)	.01^b^	0.11	Medium
Campus (TUN vs TUC)	−73.53 (49.09; −171.76 to 24.70)	.14	0.04	Small
Full model	—^c^	.09	0.14	Small-medium

a The analysis included 64 participants due to listwise deletion of 1 case with a missing predictor value.

b *P*<.05 indicates statistical significance.

c Not available.

In the second model predicting total minutes of AI use (n=64), active use was the only significant predictor (adjusted β=108.42, 95% CI 23.11-193.74; *P*=.01), representing a medium effect size (Cohen *f*²=0.11), indicating that students classified as active users spent approximately 108 more minutes using AI over the study period compared with passive users. Year of training, age, and campus were not significant predictors (all *P*>.10). The full model explained 13% of the variance in total minutes of AI use (*R*²=0.13, Cohen *f*²=0.14).

### Multilevel Models

Table 6 describes the multilevel model predicting percentage of study time spent using AI (n=248 diary entries from 59 students). In this model, preclinical students reported significantly lower AI use than clinical-phase students (β=−11.30, 95% CI −21.08 to −1.51; *P*=.02), consistent in direction and significance with our primary regression in Table 4. Active use, age group, and campus were not significant predictors (all *P*>.10). The intraclass correlation coefficient (ICC) was 0.47 (95% CI 0.34‐0.60), indicating that slightly under half of the total variance in percentage of AI use was attributable to stable between-student differences, while just over half reflected fluctuation within students across their own diary entries.

**Table 6.** Multilevel (mixed effects) models of AI use across repeated diary entries among medical students at Touro University California (TUC) and Touro University Nevada (TUN), April-May 2025^a^.

Predictor	Percentage of study time using AI (n=248 entries, 59 students)^b^		Total minutes using AI (n=253 entries, 59 students)^c^	
	β (95% CI)	*P* value	β (95% CI)	*P* value
Year of training (MS1 or MS2 vs MS3, MS4, or research ref.)	−11.30 (−21.08 to −1.51)	.02^d^	−8.44 (−28.79 to 11.92)	.42
Age group (y; >27 vs <27)	0.87 (−6.77 to 8.50)	.82	−0.42 (−16.52 to 15.69)	.96
Active use (vs passive)	−0.18 (−7.59 to 7.23)	.96	10.89 (−4.68 to 26.46)	.17
Campus (TUN vs TUC)	−6.43 (−15.53 to 2.67)	.17	−9.67 (−28.63 to 9.29)	.32
Constant	30.87 (20.21 to 41.54)	<.001^d^	35.92 (13.57 to 58.28)	.002^d^

a Models include a random intercept per student (|| sid:) to account for repeated diary observations within individuals. Reference categories are clinical years (MS3, MS4, or research), passive use, age <27 years, and TUC campus. The likelihood-ratio test compares the mixed model to standard linear regression without a random intercept; a significant result confirms that within-person correlation across diary entries is present and that a multilevel approach is appropriate.

b Random effects: between-student variance: 136.88 (83.69-223.86); within-student (residual) variance: 156.38 (128.04-191.00). Intraclass correlation: 0.47 (95% CI 0.34-0.60). Likelihood-ratio test: *χ*²_1_=73.27; *P*<.001.

c Random effects: between-student variance: 657.70 (423.59‐1021.20); within-student (residual) variance: 540.72 (444.47‐657.79). Intraclass correlation: 0.55 (95% CI 0.43-0.66). Likelihood-ratio test: *χ*²_1_=120.09; *P*<.001.

d *P<.*05.

In the parallel model predicting AI minutes per diary entry (n=253 entries from 59 students; note this outcome is defined at the diary-entry level rather than as the person-level total used in Table 5), none of the fixed effects reached significance (all *P*>.10). The direction of the active-use effect was consistent with our original aggregated model, but the association did not remain significant when examined at the diary-entry level (*β*=10.89, 95% CI −4.68 to 26.46; *P*=.17). The ICC for this outcome was 0.55 (95% CI 0.43‐0.66).

An exploratory model additionally including diary wave (entry 1‐7) as a fixed effect found no significant linear trend in AI use across the 21-day period (all *P*>.05), suggesting day-to-day fluctuation rather than systematic drift over time.

To further characterize this variability, we calculated each student’s own standard deviation in percentage AI use across their diary entries. Among the 53 students with or more valid diary entries, the mean within-person SD was 10.6% (SD 8.2%; range 0%‐28.4%; Table S1 in Multimedia Appendix 2), indicating substantial day-to-day fluctuation within individual students that would not be visible using person-aggregated scores alone (Figure S1 in Multimedia Appendix 2).

## Discussion

### Principal Findings

This pilot study found that medical students used AI tools during 19% (35.8 min) of their total study time. Clinical-phase students (MS3-MS4) were more likely to use AI for studying than preclinical students (MS1-MS2), and students who engaged actively with AI spent more total time using these tools. Overall, AI use was primarily passive, with students relying on tools such as ChatGPT and Google Gemini to simplify concepts, answer practice questions, and generate summaries. AI use varied considerably from day to day within the same student, with roughly half of the differences we observed in AI use reflecting within-person fluctuation rather than stable differences between students. This suggests that a student’s AI use is less a fixed trait and more likely something that shifts with daily demands, such as upcoming exams, assignment deadlines, or workload.

Clinical-year students were more likely to use AI for practice questions, likely reflecting preparation for licensing exams such as Comprehensive Osteopathic Medical Achievement Test (COMAT) and USMLE Step 2 CK during the study period. These students may also face greater time pressure during rotations and use AI as an on-demand resource for rapid review. The higher proportional use among clinical students may also reflect greater familiarity with AI tools through clinical exposure and a curriculum that offers fewer structured study resources compared to didactic years.

Students classified as active AI users spent more total time using AI, likely due to iterative questioning, critical evaluation, and integration into study or clinical reasoning tasks. In contrast, passive use tends to involve brief, one-directional consultation [22]. When we looked more closely at individual diary entries rather than each student’s overall total, the data became more nuanced. Total AI minutes appeared higher among active users when we summed across their entire 3-week diary period. However, this advantage was not visible when comparing single diary entries to one another. Active users may have used AI more consistently over the study period and submitted more diary entries rather than spending significantly more time on AI in any single sitting. This is helpful for thinking through “active use” patterns. Active users are not necessarily more intensively engaged each time they sit down to study, but could instead engage with AI tools more frequently or more consistently over time.

Our data do not capture whether students verified or critically evaluated AI outputs. The predominance of passive use patterns in our sample raises questions about whether students are using critical thinking to safely use AI in clinical contexts. This is consistent with findings from other studies suggesting that many students do not routinely check AI outputs against authoritative sources [23]. This gap represents an important area for curriculum development and institutional guidance.

### Comparison With Prior Work

Although there are some data that suggest that AI tools may improve efficiency and personalization, evidence on their impact on deep learning remains mixed. Some studies suggest AI use may hinder conceptual understanding, synthesis, and application of knowledge and raise concerns about the misinterpretation or acceptance of inaccurate outputs [5,22]. One study of accounting students found lower exam performance associated with ChatGPT use [5]. Given that most use in our study was passive, the implications for learning outcomes and cognitive offloading warrant further investigation.

To date, no prior studies have quantified AI use as a proportion of total study time. A 2023 US survey of 415 medical students found that 52% reported using ChatGPT for schoolwork. International studies of self-reports vary widely: 42% in Sudan, 62% in China, and nearly 90% in Palestine [6-8]. A global survey of 3839 college students found that 86% reported using AI for studying [24], suggesting continued growth in adoption.

In our study, 61% (n=231) of the students supported formal integration of AI into curricula. Early educational interventions show promise: the extracurricular AIM program improved AI literacy among premedical students [25], and a 14-week randomized trial of a ChatGPT-based coaching tool demonstrated gains in self-directed learning and critical thinking [26]. Other tools have shown more modest effects [27]. These findings suggest that carefully designed AI tools may enhance learning.

### Limitations

This study has several limitations. Self-reported data, even with a diary methodology designed to reduce recall bias, remain subject to social desirability bias and time estimation error; self-reported minutes may not precisely reflect actual usage, and triangulation with device-level data was not possible.

A standardized, validated framework for classifying active versus passive AI use does not yet exist. Recent commentary similarly argues that this binary may be too coarse to capture the complexity of student engagement with AI [20], with related scholarship proposing more graduated constructs such as students’ epistemic agency [21]. Our classification should therefore be understood as a provisional, formative operationalization rather than a validated instrument.

Our nonrandom convenience sample from 2 osteopathic schools, combined with a 5.3% (n=71) response rate, limits generalizability. We cannot rule out self-selection toward students more engaged with or curious about AI, which would tend to inflate our estimates of AI adoption and usage intensity; the findings should be interpreted as characterizing a self-selected subgroup rather than the broader student population at either institution.

The modest sample size (n=71) reduced power for subgroup analyses, reflected in wide CIs. Supplementary mixed-effects models using diary-level data (Table 6) confirmed that within-person fluctuation accounted for roughly half of the total variance in AI use (ICC=0.47‐0.55) and corroborated our primary training-year finding, though the diary-level active-use association did not reach significance, likely reflecting reduced precision at this more granular level.

Finally, our 3-week study period (April-May) may not capture AI use patterns at other points in the academic calendar. We lacked a validated digital literacy measure and did not assess academic performance or learning outcomes, limiting the interpretation of our findings for curricular recommendations. Unmeasured confounders, such as AI proficiency and baseline academic performance, may also have influenced results.

### Conclusions

Although these findings are preliminary and should be interpreted with caution, they suggest that medical students are incorporating AI into a substantial proportion of their study time. Distinct patterns by training stage and engagement style were observed in this pilot cohort. Because our study did not measure learning outcomes, we cannot draw conclusions about whether AI use is beneficial or harmful. Given mixed evidence regarding the impact of AI on deep learning and potential risks related to uncritical acceptance of AI-generated content, these findings highlight the need for further research on learning outcomes. Institutions may wish to consider providing guidance on responsible AI use, including critical evaluation, verification of outputs, and integration into evidence-based study strategies. Future studies should include larger, more diverse samples; validated digital literacy and outcome measures; and analytical designs that exploit the longitudinal structure of diary data. The digital diary approach used in this study offers a practical method for capturing real-time AI use and may be useful for evaluating future educational interventions.

## Supplementary material

10.2196/96895 Multimedia Appendix 1 Digital diary questionnaire.

10.2196/96895 Multimedia Appendix 2 Within-person variability in percentage of study time using AI across diary entries (n=53 students with ≥2 valid diary entries).

10.2196/96895 Checklist 1 STROBE checklist for cohort studies.

## Abbreviations

CITI: Collaborative Institutional Training Initiative
COMAT: Comprehensive Osteopathic Medical Achievement Test
DO: doctor of osteopathy
HIPAA: Health Insurance Portability and Accountability Act
ICC: intraclass correlation coefficient
IRB: Institutional Review Board
MCAT: Medical College Admission Test
STROBE: Strengthening the Reporting of Observational Studies in Epidemiology
TUC: Touro University California
TUN: Touro University Nevada
USMLE: United States Medical Licensing Examination
