# Supplementary material for: AI Tool Use Among Osteopathic Medical Students: Pilot Digital Diary Study
Source: JMIR Form Res. 2026 Sep 1;10:e96895. doi: 10.2196/96895 (PMC13533313; doi:10.2196/96895)
Supplement: Supplementary file 1 [file formative-v10-e96895-s001.pdf]

Digital Diary

1. Please enter your cell phone number? (Please enter the same number indicated in your intake survey and informed consent).
    a. Input: XXX-XXX-XXXX
2. Did you use any AI tools for studying or school work in the past 48 hours?
    a. Y/N

3. Which of the following AI tools did you use today - Select All That Apply?
    a. ChatGPT, Google Gemini, AMBOSS AI, UpToDate Clinical AI, other

4. In which of the following ways did you use any AI tools: - Select All That Apply?
    a. Learning more about complex medical concepts
    b. Getting simplified explanations of difficult concepts/topics
    c. Generating mnemonic/s or memory aids
    d. Creating study plans or schedules
    e. Summarizing lecture notes or textbooks
    f. Interpreting lab results or imaging findings
    g. Simulating patient interactions or clinical scenarios
    h. Generating practice questions for exams like the USMLE/COMLEX?STEP/COMAT
    i. Using spaced repetition or active recall techniques
    j. Generating clinical flowcharts or algorithms for treatment planning
    k. Creating summaries of diseases and treatments
    l. Answering practice questions
    m. Completing assignments for class

5. What percentage of your total studying/school work time today was spent using (or being assisted by?) AI?
    a. %

6. Are you currently in a “testing period” (1 week leading up to an in-house exam or a dedicated period preparing for board exams like USMLE Step 1/2, COMLEX 1/2, etc.)?
    a. Yes/ No

7. Are you currently on a break from school or rotations (school is not in session at this time, ie: Spring Break)?
    a. Yes/ No

8. Approximately how many minutes did you spend studying in the last 48 hours?

    a. 0–60 minutes, 61–120 minutes, 121–180 minutes, 181–240 minutes, More than 240 minutes

9. Approximately how many minutes did you spend using any AI tools for studying in the last 48 hours?
    a. 0 minutes, 1–30 minutes, 31–60 minutes, 61–90 minutes, More than 90 minutes
