# Supplementary material for: AI Tool Use Among Osteopathic Medical Students: Pilot Digital Diary Study
Source: JMIR Form Res. 2026 Sep 1;10:e96895. doi: 10.2196/96895 (PMC13533313; doi:10.2196/96895)
Supplement: Supplementary file 3 [file formative-v10-e96895-s002.docx]

**Supplementary Table 1.** Within-person variability in percentage of study time using AI across diary entries (n=53 students with ≥2 valid diary entries)

| **Statistic** | **Value** |
| --- | --- |
| Mean within-person SD (% AI use) | 10.6 |
| SD of within-person SDs | 8.2 |
| Range of within-person SDs | 0.0–28.4 |
| Mean diary entries per student (full completer sample, n=65) | 3.8 (SD 2.2) |

*Note: Of the 65 students who submitted at least one diary entry, 53 (82%) had two or more valid entries for percentage AI use and were included in within-person SD calculations; the remainder had a single valid entry or had all entries excluded due to missing minutes data or the AI-time-exceeds-study-time data quality flag described in Methods.*

*Supplementary Figure 1*
